# Correlation between Power Elbow Flexion and Physical Performance Test: A Potential Predictor for Assessing Physical Performance in Older Adults

**DOI:** 10.3390/jcm12175560

**Published:** 2023-08-26

**Authors:** Sergi Rodríguez-Rodríguez, Esther Jovell-Fernández, Leonor Cuadra-Llopart, Jacobo Rodríguez-Sanz, Noé Labata-Lezaun, Carlos López-de-Celis, Joan Bosch, Albert Pérez-Bellmunt

**Affiliations:** 1 Department of Basic Sciences, Faculty of Medicine and Health Sciences, Universitat Internacional de Catalunya, 08195 Barcelona, Spainaperez@uic.es (A.P.-B.); 2 Actium Functional Anatomy Research Group, Sant Cugat del Vallés, 08195 Barcelona, Spain; 3 Department of Medicine, Faculty of Medicine and Health Sciences, Universitat Internacional de Catalunya, 08195 Barcelona, Spain; 4 Department of Epidemiology, Consorci Sanitari de Terrassa, 08227 Terrassa, Spain; 5 Department of Geriatric Medicine, Consorci Sanitari de Terrassa, 08227 Terrassa, Spain; 6 Physiotherapy Department, Faculty of Medicine and Health Sciences, Universitat Internacional de Catalunya, 08195 Barcelona, Spain; 7 Fundació Institut Universitari per a la Recerca a l’Atenció Primària de Salut Jordi Gol i Gurina (IDIAPJGol), 08007 Barcelona, Spain

**Keywords:** older adults, muscle power, physical performance, correlation, functional tests

## Abstract

Background: With the increasing number of older adults and their declining motor and cognitive function, it is crucial to find alternative methods for assessing physical functionality. The Short Physical Performance Battery (SPPB), the Time Up and Go (TUG) test, the 4 Meter Walk Test and the Barthel Index (BI) have been used to evaluate mobility and fragility and predict falls. But some of these functional test tasks could be difficult to perform for frail older adults or bedridden patients that cannot ambulate. This study aimed to evaluate the relationship between these functional tests and the power elbow flexion (PEF test). Material and methods: A correlation study was designed with 41 older adults over 65 years of age. The upper limb muscle power was measured using a linear encoder (VITRUBE VBT) with the flexion of the elbow. Results: Strong correlations were found between the PEF test and the 4mWT (rho = 0.715, *p* = 0.001) and TUG (rho= −0.768, *p* = 0.001), indicating that the greater the upper limb muscle power is, the greater physical performance will be. Moderate correlations were also found between the PEF and Barthel Index (rho = 0.495, *p* = 0.001) and SPPB (rho = 0.650, *p* < 0.001). Conclusions: There is a strong correlation between PEF and the functional tests, proving that older adults that have greater upper limb muscle power have better physical performance. Upper limb muscle power and PEF could be an interesting tool for the assessment of physical performance in bedridden older adults.

## 1. Introduction

According to the scientific literature, in many countries older adults are defined as having a chronological age of over 65 years [1]. The number of men and women reaching the age of 90 or older is increasing rapidly in Europe and in other developed countries. This development has led to an increase in research aimed at understanding the nature of the aging process [2]. Older adults experience a decline in motor function [3], including muscle weakness, slower gait speed and poor balance control [4]. 

Such loss in physical performance related to aging is a critical issue because this decline limits older adults’ activities of daily living (ADL) and instrumental activities of daily living [4,5], as well as contributing to increased disability, risk of falls or lower quality of life [4,6]. 

For this reason, the assessment of mobility status is needed to prevent further deteriorations in functionality as early as possible [7]. In this line, several studies have examined a variety of motor functions and physical measures of performance in relation to disability and risk of falls [8].

Scores on functional tests such as the Short Physical Performance Battery (SPPB) or the Gait Speed Test have been shown to be associated with a risk of falls, disability and even mortality [9]. The Time Up and Go (TUG) test objectively measures function and mobility and dynamic balance in older adults [10,11]. Gait speed and its valorization with the 4 Meter Walk Test (4mWT) are among the most widely used measurements of functional mobility and performance of activities of daily living, especially for older adults [12], and one of the most commonly used methods to evaluate walking speed in clinical geriatric settings [13]. The Barthel Index (BI) focuses on self-care and mobility, giving older people autonomy and independence to live without needing help from other adults [14]. These functional tests are often useful in the clinical field, and experts recommend screening all older adults for physical performance in primary care to detect those at risk for frailty and/or sarcopenia [15].

However, the valuable information from some of these functional tests (such as SPPB, 4mWT or TUG) could be difficult to collect for some frail older adults or bedridden patients who cannot ambulate. Moreover, recent studies have described how frail older adults have various deficiencies and cannot adequately compensate to maintain an optimal functional level [16].

For these reasons, previous research [17] has reported that handgrip strength can be considered a health biomarker, mainly due to its association with overall muscle strength in healthy people and with different pathologies. In addition, its use has been included as a tool for the early detection of chronic diseases in multiple international scientific investigations [18]. On the other hand, muscle power has recently been shown to be positively associated with the ability to perform activities of daily living, and may be a stronger predictor of functional dependency than muscle strength [19]. But considering the impairments of some older adults, we have to question the measurement of leg muscle power, since as has been observed in one of the few large epidemiological studies evaluating muscle power among older adults, impairments in leg muscle power were found to impart a greater likelihood for significant mobility limitations than impairments in leg strength [19].

Moreover, in addition to the described importance of muscle power, a substantial association between upper and lower limb power has been found to exist, suggesting that muscle power may depend on a physiological attribute. This may reflect aging vulnerable neuromuscular mechanisms underlying movement speed, such as muscle fiber type and contractile properties, motor unit synchrony and firing time, muscle movement capacity and muscle movement capacity contractile properties, synchrony and firing time of the motor units, the control of agonist and antagonist muscle groups and nerve conduction velocity [20]. The results of this investigation suggest that upper limb power may be an appropriate surrogate for lower body muscle power, as elbow extension is a relatively easy task to perform for the majority of older adults regardless of their health and mobility status and, from an engineering perspective, may facilitate the development of a simpler device than required to evaluate lower extremity muscle actions [21].

In line with what was described above and among the many methods used to assess muscle power, a linear encoder of traction cables has recently emerged [22,23]. Linear encoders are widely accepted in sports science to measure sports performance using displacement over time [24]. However, authors such as [25] have described that they are rarely used to assess physical performance in older people. Nevertheless, power has become an essential predictor of functionality in older men and women [26,27].

The scientific literature has shown the functional and cognitive changes suffered by older adults during old age, which may lead to frailty or disability. These changes are in many cases evaluated by functional assessment scales, but in frail older adults this is very difficult due to their low functional level, and that in many cases they are bedridden and cannot walk. Added to this, previous studies have verified how, due to deficiencies in the lower extremities, assessing muscle power in the lower extremities, like in most studies, can be a difficult task in some older adults. In this case, and knowing that today linear encoders allow for the measuring of muscle power in older adults, a suitable upper limb measurement can serve as a surrogate measure for lower limb muscle power measurements. This study aims to evaluate whether there is a correlation between the functional tests and the PEF, in order to establish new functionality predictions in frail or hospitalized older adults. A secondary objective is to compare PEF and grip strength, to evaluate which of the two variables presents a stronger association with the categorical classification of the functional tests.

## 2. Materials and Methods

### 2.1. Study Design

A cross-sectional observational correlational study was performed, following the Strengthening the Reporting of Observational Studies in Epidemiology (STROBE) statement.

### 2.2. Sample Size Calculation 

The GRANMO v 7.12 program was used to calculate the sample size. A correlation coefficient analysis was performed with an alpha risk of 0.05, bilateral contrast, beta risk of 0.20, a moderate Pearson correlation coefficient estimate of 0.4 and a loss forecast of 10%, and a necessary sample of 41 subjects was obtained.

### 2.3. Participants

The sample consisted of older adults living in long-term care facilities with or without functional dependence or cognitive decline. Participants and their families or legal guardians were verbally informed and agreed to participate in this study, signing their informed consent. The participants were from an old age home (Terrassa, Barcelona) and a nursing home (Matadepera, Barcelona). The measurements were carried out by the investigators in Terrassa and Matadepera between January 2023 and July 2023.

Inclusion criteria were (a) people over 65 with or without musculoskeletal disorders and cognitive declines. Exclusion criteria were (a) having been in a similar study before, (b) having a bone fracture in the previous six months, (c) having uncontrolled symptomatic cardiovascular or respiratory disease, and (d) having an inability to understand the information provided by the assessors.

### 2.4. Outcomes

The primary variable was the upper extremity power with linear encoder and handgrip strength, and the secondary variables of physical performance were recorded through the SPPB, the 4 Meter Walk Test (4mWT), the Barthel Index (BI) and the Timed Up and Go Test (TUG).

#### 2.4.1. Power Elbow Flexion (PEF) 

**Figure 1:**
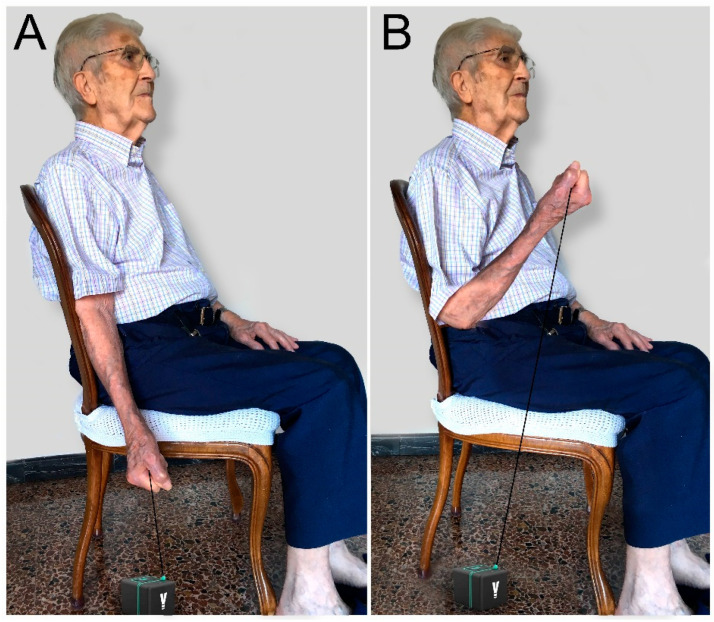
Power elbow flexion (PEF test) procedure. The procedure consists of assessing the power of elbow flexion using a linear encoder. For this, the subject was seated (**A**) and flexed the elbow as quickly as possible (**B**).

The PEF test was measured with the linear encoder (VITRUBE VBT). Muscular power was evaluated during the elbow flexion movement (Figure 1). It was performed 5 times and the average of all the repetitions was calculated to obtain the result. The unit of measurement used was watts. 

#### 2.4.2. Short Physical Performance Battery (SPPB)

This assessment battery comprises three distinct tests: a balance assessment, a four-meter walking speed test and a five-repetition chair sit-to-stand task. Each test yields a numeric score ranging from 0 to 4, and these scores are aggregated to establish a composite score spanning 0 to 12. The test–retest reliability of the battery has demonstrated a favorable to outstanding range (Intraclass Correlation Coefficient–ICC: 0.83–0.92). Moreover, the inter-rater reliability among older adults admitted on an acute basis was determined to be exceptional (ICC: 0.91) [28]. Based on this study [29], a categorization of the numerical results of the SPPB test was carried out to make a correlation with the upper limb muscle power. SPPB total scores range from 0 to 12 points: 0–3 points (disabled/very low performance), 4 to 6 points (poor performance), 7 to 9 points (moderate performance), and 10–12 points (good performance).

#### 2.4.3. The 4 Meter Walk Test (4mWT)

This is a functional test that reflects the average speed at which the subject walks 4 m. Although it is included in the SPPB battery, its score has a value by itself. Its reliability has been previously studied (ICC = 0.96, 95%CI = 0.94–0.98; SEM = 0.01) [30]. Based on the following study [31], a categorization of the numerical results of the 4mWT was carried out to make a correlation with the upper limb muscle power. Slow gait speeds (<0.97 m/s) are associated with frailty, and (<1.0 m/s) suggests no frailty.

#### 2.4.4. Timed Up and Go Test (TUG)

This is a functional test that reflects the time in seconds that it takes the person to get up from the chair, with the help of the arms, walk 3 m, turn around an obstacle, return to the chair and sit down again (15). Its reliability has been studied previously (ICC = 0.98, 95%CI = 0.93–1.00; SEM = 0.7) [28]. Based on the following study [32], a categorization of the numerical results of the TUG test was carried out to make a correlation with the upper limb muscle power. Less than 10 s: low risk of falling. Between 10 and 20 s: risk of falling. More than 20 s: high risk of falling.

#### 2.4.5. Barthel Index (BI)

The BI is an ordinal scale that measures the actual performance of 10 basic activities of daily life (ADLs), such as dressing, mobility, and grooming, in the domain of activities of the International Classification of Functioning, Disability and Health. Its reliability has been studied previously (ICC = 0.96, 95%CI= 0.93–0.98; SEM = 1.1) [33]. Based on the study [34], a categorization of the numerical results of the Barthel Index was carried out to make a correlation with upper limb muscle power: (0–20) total dependency; (21–60) severe dependency; (61–90) mild independency; (91–99) moderate independency; and (100) independency.

#### 2.4.6. Handgrip Strength

This is a test that shows the maximal grip strength in kilograms (Kg) using a hand dynamometer. The device used was the Jamar^®^ dynamometer (Lafayette Instrument Company, IN, USA). The subject was placed in a seated position with the arms supported, ensuring 90° elbow flexion with the wrists in a neutral position. Three measurements were taken for both the dominant and non-dominant arms, with a one-minute rest between measurements. The mean between the three measurements of each hand was calculated and the hand that obtained the best results was chosen. The validity and reliability of this device has been evaluated in previous studies (ICC = 0.98) [35].

### 2.5. Statistical Analysis

For the statistical analysis, the software SPSS (IBM SPSS Statistics for Windows, Version 26.0. Armonk, NY, USA: IBM Corp), was used. The variables assessed were upper limb muscle power, handgrip strength, SPPB, 4MWT, TUG and Barthel index. Descriptive statistics (mean and standard deviations, or number and percentage) were calculated to describe sample characteristics. The normal distribution of the variables was analyzed using the Shapiro–Wilk test. Correlation analysis was performed by using Spearman’s rank correlation coefficient. The following intervals were used in order to interpret the strength of the correlation coefficient: 0–0.10, negligible correlation; 0.10–0.39, weak correlation; 0.40–0.69, moderate correlation; 0.70–0.89, strong correlation; and 0.90–1.00, very strong correlation.

Subsequently, a one-factor ANOVA was performed, taking the cut-off values according to each variable (SPPB, 4MWT, TUG, Barthel index) to see the association with the upper limb muscle power and handgrip strength, and Bonferroni post hoc. The significance level was set at *p* < 0.05.

## 3. Results

The baseline characteristics of the 41 participants are shown in Table 1. As shown, the mean age was (82.0 ± 9.6), with a mean height of 155.2 ± 9.1 and a mean weight of 66.5 ± 11.8. As can be seen in the table, almost the entire sample (95.1%) presented right dominance in the upper extremity.

Table 2 shows the data obtained from the different functional tests and the upper limb measurements with the linear encoder, using the mean and standard deviation, together with the categories of each functional test and the sample or percentage used. The mean power for the participants was 398.4 ± 291.6 watts. For the Barthel Index, the mean punctuation was 84.4 ± 18.4 points and almost half of the participants were mildly dependent (48.8%). For the handgrip strength test, the mean punctuation was 11.5 ± 8.4 KG. For the SPPB, the mean punctuation was 6.8 ± 4.4 points. We found that 13 participants (39.4%) were classified as “without limitations” and 13 patients (39.4%) had severe limitations. For the 4mWT, the mean punctuation was 0.8 ± 0.6 m/s. A percentage of 75.5% of the participants had fragility based on the categories. For the TUG, the mean punctuation was 22.2 ± 13.3 points and we can see that half of the participants (48.7%) were classified as at high risk of falling.

A study of correlations through Spearman’s Rho was carried out between the upper limb muscle power measured with the linear encoder and handgrip strength, with the quantitative values of the variables Barthel, SPPB, gait speed (4mWT) and TUG. Subsequently, an ANOVA analysis of one factor was performed to see if the upper limb muscle power measured with the linear encoder and handgrip strength was associated with the degree of functional independence or frailty of the categorical variables (SPPB, 4MWT, TUG, Barthel index). The results of the ANOVA analysis of variance are shown in Table 3.

### 3.1. Barthel Index

For the Barthel Index, moderate statistically significant positive correlations were found with the encoder power (rho = 0.495, *p* = 0.001), and weak ones with the handgrip (rho = 0.382, *p* = 0.023). This variable was divided into five categories: 0–20, total dependency; 21–60, severe dependency; 61–90, moderate dependency; 91–99, low dependency; and 100, independency [34]. An association was found between the Barthel categorical variable and the encoder (*p* = 0.011), and also with the handgrip (*p* = 0.006). In the post hoc analysis, we found that there was a statistically significant difference between the independent and mildly dependent categories for the Encoder (*p* = 0.033), and also for the handgrip (*p* = 0.007).

### 3.2. SPPB 

For the SPPB, moderate statistically significant positive correlations were found with the power of the encoder (rho = 0.650, *p* < 0.001), and with the handgrip (rho = 0.530, *p* = 0.004). This variable was divided into four classes: 0–3 points (disability/very low performance), 4–6 points (low performance), 7–9 points (moderate performance) and 10–12 points (good performance) [29]. An association was found between the categorical variable SPPB and the encoder (*p* = 0.010), but not with the handgrip (*p* = 0.066). In the post hoc analysis, we found that there was a statistically significant difference between the categories ‘without limitation’ and ‘severe limitation’ (*p* = 0.006).

### 3.3. The 4mWT

For the 4mWT gait speed variable, strong statistically significant positive correlations were found with the encoder power (rho = 0.715, *p* = 0.001), and moderate ones with the handgrip (rho = 0.663, *p* < 0.001). This variable was divided in two categories: <0.97 m/s (fragility) and >1.0 m/s (no fragility) [31]. A statistically significant association was found between the categorical classification of frailty (*p* = 0.010) with the encoder, and also with the handgrip (*p* < 0.001). 

### 3.4. TUG

For the TUG, strong statistically significant negative correlations were found with the power of the encoder (rho= −0.768, *p* = 0.001), and moderate ones with the handgrip (rho = 0.6240, *p* < 0.001). This variable was divided into three categories: <10 s (low risk of falling), 10–20 s (risk of falling) and >20 s (High risk of falling) [29]. An association was found between the categorical variable TUG and the encoder (*p* = 0.001), and also with the handgrip (*p* < 0.001). In the post hoc analysis, we found that there was a statistically significant difference between the no risk of falling and moderate risk of falling (*p* = 0.005) and the no risk of falling and serious risk of falling (*p* < 0.001) categories, with the encoder. With the handgrip, it was found that there was a statistically significant difference between no risk of falling and high risk of falling (*p* < 0.001) and moderate risk of falling and high risk of falling (*p* = 0.046).

## 4. Discussion

The present study aimed to evaluate the correlation between the PEF and handgrip strength with different functional tests in older adults. The main results of this study show strong correlations between the PEF and the TUG and 4mWT and a statistically significant association between the PEF and the categorical classification of all the functional tests. 

It is well known from the literature that, during the process of aging, there are changes that worsen the state of health and physical fitness, causing a deterioration in organic functions such as physical, psychological and social functionality [36]. Frailty develops in older people, increasing the risk of adverse events such as functional impairment, dependency and falling, and is considered a biological condition in which there is a poor response by several physiological systems to maintaining homeostasis after a stressful event [37].

Over the last few years, investigations have found different tools such as the Walking Speed or the Time Up and Go (TUG) for predicting frailty, physical performance and the risk of falls in older adults [14]. Moreover, the Barthel Index has been widely used to measure the subject’s level of dependency [38], as well as the SPPB test to identify declines in physical performance and physical frailty [39]. Despite increasing evidence [40] of the benefit of assessing frailty to provide optimal decision-making, the common approaches to identifying frailty are limited. Most of them are clinically cumbersome and time consuming or are based on gait-centered measures, which are not useful for mobility-impaired individuals. In other cases, making the patient walk or perform these tests is not feasible due to motor deficits in the lower extremities or the patient being bedridden. In our study, the assessment of the PEF could be carried out and extrapolated to this type of subject (bedridden or unable to ambulate).

On the other hand, current evidence positions handgrip strength as a reliable marker for total muscle strength for older adults [41], and it is considered an important vitality surrogate for general fitness, cognitive status, frailty and sarcopenia in older adults [42]. In our study, the handgrip strength had moderate correlations with the SPPB test, TUG and 4mWT, and also had a statistically significant association with the degree of independence functional or frailty of the categorical variables of TUG, 4mWT and SPPB. These results are consistent with previous studies on the reliability of the handgrip as a predictor of health in older adults.

However, and taking into account the aim of our study, new evidence has shown that muscle power is an essential aspect of many daily living activities and declines faster than other fitness parameters. In old investigations, it was described that many muscle power measures such as jumping are contraindicated for use with many older adults [43]. At present, some studies have found that leg extension power is highly positively associated with the functional performance tests [44], and even better, they found strong associations between upper and lower body muscle power among mobility-limited older adults, indicating that upper limb muscle power has validity as a measure of muscle power to address the mobility problems of older adults [21]. Furthermore, another study with frail older adults (>60) developed an upper-extremity function assessment method based on power elbow flexion that was significantly associated with the frailty categories of the gold standard, the Fried Index [45].

In this line and among the many methods used to assess muscle power, the linear encoder, which is reasonably inexpensive, portable, and easy to apply, has recently become available [46]. In our study, we used a linear encoder to measure the upper limb muscle power of the older adults, to see if there was a correlation with functional performance tests, as was carried out in this study [44], but with leg muscle power. In concordance with the scientific evidence, our study found strong correlations between upper limb muscle power and TUG and 4mWT. In addition, and in line with our results, this study [47] also demonstrated a significant association between the upper extremity function based on power elbow flexion and the fall risk, and between the power elbow flexion and gait speed (r = 0.68, *p* < 0.001).

Moreover, and clinically importantly, we found that when we compared the correlations of the linear encoder with the functional tests and the correlations of the handgrip with the same functional tests, the upper limb muscle power measured with the linear encoder presented better correlations with all the functional tests than the handgrip strength. These results, even taking into account the extremity assessed, could be in line with the study of [48], where they found that lower extremity muscle power was more closely related to physical performance than muscle strength. Kozicka et al. [49] also found a stronger correlation in quadriceps muscle power than handgrip strength in older institutionalized adults.

In our study, the upper limb muscle power measured with the linear encoder had a stronger association with the categorical variables of TUG and SPPB tests than the Handgrip strength. We found that the upper limb muscle power, measured with the linear encoder, could better discriminate frail from non-frail patients in the SPPB and TUG tests than handgrip strength. In fact, handgrip strength did not present any association with the categorical variables of SPPB. Our results agree with the study of Kozicka et al. [50], which found a correlation between muscle power and functional tests such as ADLs, the TUG test, the Tinetti test, and the 6MWT (6 min walking test). Moreover, our results also agreed with another study [50], where they found significant associations between upper-extremity functional tests based on elbow flexion–extension (UEFI) and functional mobility tests, suggesting that upper extremity function may provide a comparable marker of physical frailty. These results highlight that, as many ADL require the ability to perform short, intensive exercises, which demand appropriate muscle power, functional abilities depend primarily on how quickly the muscles can generate strength, not only on how strong they are [51]. In that sense, the present study shows that upper limb muscle power, measured with the linear encoder, could be an interesting tool to identify physical performance impairments, frailty and potential risks of falls in older adults.

Our study has some limitations. To present a sample that is as real as possible (to be helpful in clinical practice), people with and without functional and cognitive deficits have been included. Indeed, with a more uniform sample, these results would have been even more powerful. In the present study, only the patient’s dominant upper extremity has been evaluated; we are still determining the results that would be obtained with the other extremity. It may be engaging in future lines to assess predictive variables, so multivariate correlational studies would be interesting.

## 5. Conclusions

There is a strong correlation between the PEF test with the TUG and 4mWT, and a moderate correlation between this test with the Barthel Index and SPPB. These results show that greater muscle power in the elbow flexion provided better physical performance in adults over 65 years of age in our study. Even so, it should be noted that in future research the weight of the dominant upper extremity should be taken as a reference to obtain specific values and be able to make an adequate correlations. 

Statistically significant associations between the upper limb muscle power and the categorical variables of all the functional tests were found. These results can suggest to us that the PEF test measured with the linear encoder could be a potential predictor of functionality and fragility in older adults. The results of our study may have an important clinical value in frail or bedridden older adults, in order to predict their functionality or the degree of frailty without the need to use functional tests.

## Figures and Tables

**Table 1:** Subjects’ demographic characteristics.

Variable	Mean ± SD or *n* (%)
Sex	
Women	35 (85.4%)
Men	6 (14.6%)
Age (years)	82.0 ± 9.6
Height (cm)	155.2 ± 9.1
Weight	66.5 ± 11.8
Dominant Limb	
Right	39 (95.1%)
Left	2 (4.9%)

Abbreviations: SD, standard deviation; *n*, number; cm, centimeter.

**Table 2:** Data on the variables under study.

Variable	Mean ± SD or *n* (%)
Encoder EF (power)	398.4 ± 291.6
Handgrip (Kg)	11.5 ± 8.4
Barthel Index (points)	84.4 ± 18.4
Barthel	
Independent	17 (41.5%)
Mildly independent	20 (48.8%)
Moderately independent	4 (9.8%)
SPPB (points)	6.8 ± 4.4
SPPB	
Good performance	13 (39.4%)
Moderate performance	5 (15.2%)
Poor Performance	2 (6.1%)
Disabled	13 (39.4%)
4mWT (m/s)	0.8 ± 0.6
4mWT	
No frailty	10 (25.6%)
Frailty	29 (75.5%)
TUG (points)	22.2 ± 13.3
TUG	
No risk of falling	7 (17.9%)
Risk of falling	13 (33.3%)
High risk of falling	1 (48.7%)

Abbreviations: SD, standard deviation; *n*, number; EF, elbow flexion; SPPB, short physical performance battery; 4mWT, 4 m walk test; TUG, time up and go, m/s, meters per second.

**Table 3:** ANOVA analysis of variance.

	Encoder			Handgrip		
Variable	*n*	Mean ± SD	*p*	*n*	Mean ± SD	*p*
Barthel						
Independent	17	549.47 ± 350 78	0.011	14	15.91 ± 9.97	0.006
Mildly independent	20	312.55 ± 187 15		18	7.29 ± 4.30	
Moderately independent	4	185.50 ± 128.44		3	16.11 ± 7.71	
	*n* = 41			*n* = 35		
SPPB						
Good performance	13	645.77 ± 367 13	0.010	11	17.81 ± 9.93	0.066
Moderate performance	5	400.00 ± 164.87		4	8.42 ± 5.10	
Poor performance	2	437.00 ± 22.63		2	13.00 ± 1.41	
Disabled	13	272.15 ± 138.82		11	8.61 ± 7.24	
	*n* = 33			*n* = 28		
4mWT						
No frailty	10	681.10 ± 400.38	0.000	8	20.30 ± 10.06	0.000
Frailty	29	308.10 ± 175.42		25	8.91 ± 5.49	
	*n* = 39			*n* = 33		
TUG						
No risk of falling	7	788.71 ± 435.38	0.000	5	21.53 ± 12.36	0.001
Risk of falling	13	434.69 ± 161.19		11	13.96 ± 6.95	
High risk of falling	19	240.74 ± 136.44		17	7.29 ± 3.99	
	*n* = 39			*n* = 33		

Abbreviations: SD, standard deviation; *n*, number; SPPB, short physical performance battery; 4mWT, 4 m walk test; TUG, time up and go; *p*, *p*-value.

## Data Availability

Not applicable.
